# Cross-Linguistic Word Recognition Development Among Chinese Children: A Multilevel Linear Mixed-Effects Modeling Approach

**DOI:** 10.3389/fpsyg.2020.00544

**Published:** 2020-04-16

**Authors:** Connie Qun Guan, Scott H. Fraundorf

**Affiliations:** ^1^Faculty of Foreign Studies, Beijing Language and Culture University, Beijing, China; ^2^Center for the Advances of Language Sciences, University of Science and Technology, Beijing, China; ^3^Department of Psychology, Carnegie Mellon University, Pittsburgh, PA, United States; ^4^Department of Psychology and Learning Research and Development Center, University of Pittsburgh, Pittsburgh, PA, United States

**Keywords:** orthographic awareness, grapheme recognition development, multilevel linear mixed models, frequency, AoA, Chinese-English bilingual children

## Abstract

The effects of psycholinguistic variables on reading development are critical to the evaluation of theories about the reading system. Although we know that the development of reading depends on both individual differences (endogenous) and item-level effects (exogenous), developmental research has focused mostly on average-level performance, ignoring individual differences. We investigated how the development of word recognition in Chinese children in both Chinese and English is affected by (a) item-level, exogenous effects (word frequency, radical consistency, and curricular grade level); (b) subject-level, endogenous individual differences (orthographic awareness and phonological awareness); and (c) their interactive effect. We tested native Chinese (Putonghua)-speaking children (*n* = 763) in grades 1 to 6 with both Chinese character and English word identification (lexical) decision tasks. Our findings show that (a) there were effects of both word frequency and age of acquisition in both Chinese and English, but these item-level effects generally weakened with increasing age; (b) individual differences in phonological and orthographic awareness each contributed to successful performance; and (c) in Chinese, item-level effects were weaker for more proficient readers. We contend that our findings can be explained by theoretical models that incorporate cumulative learning as the basis for development of item-level effects in the reading system.

## Introduction

By the end of the elementary school years, Chinese-speaking children can typically read up 2,500 Chinese characters and up to 2,000 words in English as a second language (L2 English) (NIES, 2012). Acquiring this system of lexical representations, which permits efficient word recognition, is an essential part of learning to read (Ehri, 2014; Perfetti and Stafura, 2014; Daniels and Share, 2018). In this acquisition process, mapping lexical representation to spoken words creates a foundation for lexical and phonological processing and the subsequent acquisition of new words (Perfetti and Harris, 2013). Strong associations between orthography and phonology contribute to literacy in L1 (first language) Chinese (Guan et al., 2011, 2020) and in an L2 (Gunderson et al., 2011). However, we know little about the pattern of cross-linguistic word recognition development in both L1 Chinese and L2 English among Chinese children.

In the current study, we examine variation in the cognitive reading system for L1 and L2 word recognition development among Chinese children. We track the state of the system by estimating effects on reading performance both due to critical word properties, including frequency, consistency, and age of acquisition (AoA), and due to critical child-level development variables, including phonological awareness (PA) and orthographic awareness (OA). Our study is thus the first to examine both exogenous (item-level effects) and endogenous (individual differences) variation in psycholinguistic effects during the early years of literacy in both Chinese as L1 and English as L2.

### Word Reading Development

Development reading research has employed simple tasks like word naming or lexical decision to uncover properties of the reading system in the early years of literacy acquisition. Evidence has accumulated that the average typically developing pupil is faster to respond to words that have pronunciations obeying the rules for the spelling–sound mappings of its constituent graphemes in English (e.g., Coltheart et al., 1993, 2001) or that are consistent with pronunciation of similar-looking words (e.g., Glushko, 1979; Andrews, 1982; Taraban and McClelland, 1987). Knowing what item attributes affect reading performance has motivated and constrained models about how cognitive reading processes function in English and in Chinese (Coltheart et al., 2001; Perfetti, 2007). Current theories can account for skilled reading of many languages, including both Chinese and English, and for the development of reading in English (e.g., Seidenberg and McClelland, 1989; Perfetti, 2007), but there is a need for theories that can explain reading development in languages other than English.

Thus, Davies et al. (2017) propose that developmental accounts of the reading system could be improved by observing how psycholinguistic effects vary with age. This is the challenge that we take up here. In particular, we examine two critical issues. First, although the general effects of the item-level variables mentioned above are well-established, it remains to be determined whether each of these variables also has an effect during word learning and whether these effects change with chronological age. Thus, we investigate whether item-level effects vary with grade level—or, in other words, the level of reading development.

Second, we examine whether these item-level effects are also modulated by individual differences in reading skill. Few studies have addressed both subject-level factors (such as readers’ PA and OA) and item-level variables (including frequency and other orthographic or phonological features of words or characters) together to determine whether and to what extent these two levels of variables interact.

### Item-Level Factors in Reading Development

Grapheme recognition is a hugely important skill for all children during primary school education (Shu and Anderson, 1997, 1999). Several psycholinguistic properties affect grapheme recognition, in part by affecting the ease of learning mappings between print and spoken word forms at the sublexical and lexical levels (Ho et al., 2003). Specifically, we focus on two properties of neighborhood structure, including orthography-to-phonology consistency (Taraban and McClelland, 1987) and frequency (Marslen-Wilson et al., 1994).

First, we know that oral reading in English is faster when there is a consistent mapping between orthographic representations and the corresponding phonology (Taraban and McClelland, 1987). DeFrancis (1989) has claimed that there is now little debate in English that highly consistent words are recognized quicker and more accurately. By comparison, it is generally believed that the correspondence between orthography and phonology in Chinese is more arbitrary than in English. Nevertheless, in Chinese, approximately 80% of characters afford some phonetic and semantic information (Shu et al., 2003). The phonetic radical gives a clue to the pronunciation, and the semantic radical gives a hint to character meaning. Thus, orthography-to-phonology *consistency* can be defined in Chinese as the ratio of the number of characters containing the same phonetic radical with the same pronunciation to the total number of characters containing that phonetic radical. Oral naming responses are faster and more accurate for words with high consistency (see examples under *Measures*), especially for low-frequency words, in both English (Seidenberg and Waters, 1985) and Chinese (Jared, 2002). This consistency effect has been interpreted as supporting a single mechanism for converting print into speech sounds based on statistical mappings between orthography and phonology that are learned in childhood. In particular, effects of consistency in Chinese imply that, in learning or developing the statistical mappings between orthography and phonology, orthographic similarity makes it easier to sound out individual words (Hsu et al., 2009).

Two other relevant word properties are its average AoA and frequency. We know that oral reading is faster when a word learned earlier (Cortese and Khanna, 2007) and when it is encountered more in daily usage (Marslen-Wilson et al., 1994).

Although there is consensus that each of these variables is relevant to word recognition, the developmental trajectories of their effects remain unclear. Several models of reading development (e.g., Zevin and Seidenberg, 2002; Johnston and Barry, 2006) predict that as young children’s reading experience increases, many item-level effects should diminish. For instance, Zevin and Seidenberg’s (2002) theoretical model proposes that as readers’ total reading experience accumulates, the effects of early experience (i.e., AoA) should diminish in favor of more general properties of the orthography (i.e., the consistency of the orthography-to-phonology mapping).

Indeed, Davies et al. (2017), across a variety of methods, found that frequency and AoA effects diminish with increasing age. That is, as readers grew older, their performance was less affected by how common the words are in the language or by the time point at which they learned the words. By contrast, some studies revealed similar frequency effects in younger and older readers, in studies both of children (Burani et al., 2002) and of adults (Tainturier et al., 1989; Allen et al., 1991; Cohen-Shikora and Balota, 2016). Similarly, some studies have no significant differences in the AoA effect between younger and older adults in word naming (Morrison et al., 2002; Barry et al., 2006) or lexical decision (Barry et al., 2006). Indeed, other studies have even shown a more robust frequency effect in older compared to younger adult readers (Spieler and Balota, 2000; Morrison et al., 2002; Balota et al., 2004). This has led some researchers (e.g., Morrison et al., 2002; Ghyselinck et al., 2004; Murray and Forster, 2004) to conclude that the frequency and AoA effect do not diminish with growing overall experience.

These conflicting results may in part reflect methodological differences. Specifically, Cortese and Khanna (2007) observed that the AoA effect is larger in lexical decision than in word naming, supporting the interpretation that the lexical decision task emphasizes semantics (Chumbley and Balota, 1984). Here, we use the lexical decision task with a large sample size (over 700 participants and over 180,00 trials) that should give us ample power to detect any such developmental changes.

### Interaction of Item-Level and Child-Level Factors

Our second major question was how word-level difficulty might interact with individual differences in reading skill. The *lexical quality hypothesis* (Perfetti, 1991; Perfetti and Hart, 2002) proposes that learning to read requires developing well-specified and precise phonological, orthographic, and semantic knowledge about words. Because phonology is automatically activated in character decoding (the *Universal Phonological Principle* of literacy; Perfetti and Harris, 2013), a key subject-level factor in developing these representations may be *PA*, the ability to perceive and manipulate sound units of a spoken language (Bruce, 1964; Liberman et al., 1974; Wagner and Torgesen, 1987). Evidence suggests that awareness of the phonological structure of word units plays a pivotal role in developing word representations in alphabetic orthographics, such as English (Bradley and Bryant, 1983), as well as logographic orthographies, such as Chinese, and other orthographies (Siok and Fletcher, 2001; see also Hu and Catts, 1998; Seymour et al., 2003). Indeed, PA during the preschool years plays a causal role in learning to read in the early school years (Bradley and Bryant, 1983; Treiman, 1985; Wagner and Torgesen, 1987).

Other language awareness skills are also important for developing high-quality lexical representation (Goswami and Bryant, 1990). Namely, *OA* refers to children’s understanding of orthographic conventions used in the writing system adopted in a language (Treiman and Cassar, 1997). In Chinese, OA involves knowledge of orthographic features, including the sublexical form of radicals, that convey information about character meaning. Because character neighborhoods sharing the same radical are often semantically related, awareness of radical function may be a powerful device for the acquisition of literacy in Chinese. Indeed, Ho et al. (2003) demonstrated that various types of semantic radical knowledge, including about the position and the semantic category of semantic radicals, correlate significantly with character reading and sentence comprehension. The effects of OA are not limited to Chinese; OA also explains unique variance in reading English as L1 (Berninger et al., 1991, 2010).

However, we know little about the developmental trajectories of the influences of both PA and OA across years, nor how they interact with item-level factors. Further, in the cross-linguistic context, a key question is whether the kinds of connections that children make between phonology and orthography differ depending on the phonology of the language that is being learned and the orthographic units that this phonology makes salient. Here, we investigate how the effects of PA and OA in L1 Chinese and L2 English develop across years among primary school children, as well as how these subject-level factors interact with the item-level variable of frequency.

### Present Study

Linear mixed-effects (LME) modeling permits a closer examination of these questions through item-level analysis of word and, ergo, character reading (Gilbert et al., 2011; Steacy et al., 2016). Here, we apply LME models to a large data set of lexical processing by children with Chinese characters and English words (365,760 total trials) to test item-level and subject-level factors that contribute to word recognition development in both Chinese and English. All participants are pupils from elementary schools sampled from an ongoing national-level reading assessment and intervention project in China (Guan et al., 2011, 2012, 2013, 2015, 2019). We examined the development of word recognition in children learning Chinese and English using a cross-sectional approach, examining speed and accuracy of lexical decision from the first through the sixth grade.

We applied LME modeling to examine accuracy and response time (RT) at the level of response to individual words, considering influences of both character-level properties (frequency, consistency, AoA) and subject-level properties (PA and OA), as well as the progressive change in these influences across grades. This allowed us to investigate (a) whether item-level effects on word recognition vary with age (e.g., the effects of frequency and AoA effects decrease, but consistency increases) and (b) whether item-level frequency interacts with subject-level effects. We further hypothesized that, due to limited language experience in L2, frequency might not play a role in L2 word recognition for lower graders (grades 1 to 3) and predict RT and accuracy for L2 English only for higher graders (grades 4 to 6).

We also address two limitations that may have contributed to inconsistency of results in previous studies. First, inconsistency in previous studies may result from limitations inherent in comparisons between group-level averages (e.g., of younger versus older children; Davies et al., 2017). Second, inconsistencies among previous observations may result also from limitations in the range of ages or reading abilities sampled in previous studies (typical only or atypical only). If age-related changes are confined to specific phases of development or ability, then the age ranges in which reading is tested may have a critical influence on the nature of the item effects observed. Our study addressed both limitations by examining the effect of age as a continuous variable and including all readers regardless of ability.

## Materials And Methods

### Participants

We recruited 763 students from three elementary schools in Zhejiang Province, China. All parents signed an informed consent form throughout the assessment and intervention periods from 2012 on. All participants spoke Mandarin at home as their L1.

### Measures

#### Phonological Awareness in Chinese

Participants heard a novel character pronounced and were asked to write down the pinyin and tone. The maximum score (60) was earned by producing the correct pinyin onset, rime, and tone for each of 20 characters. The reliability coefficients of this set of measures ranged from 0.81 to 0.90.

#### Orthographic Awareness in Chinese

Following Guan et al. (2015), OA was measured by testing each of stroke awareness and radical knowledge. For stroke awareness (considered a cue for retrieval of Chinese characters; Flores d’Arcais, 1994), students tried to reproduce a character one stroke at a time in what they understood to be the appropriate order A maximum score (equal to 20) was earned by writing all 20 characters using the correct stroke order. For radical knowledge, a participant was first shown a novel character and then was asked to identify the constituent radicals that could make up that novel character. For example, for character “晴,” the participants should select the appropriate constituent radicals “ 日” and “ 青 ” out of stimuli including the four semantic radicals (日, 口, 目, 月) and four phonetic radicals (青,, 亲, 庆). The maximum score (20) could be earned by correctly identifying all radicals. The scores on these two tasks were summed to produce the OA score (maximum 40 points). The reliability coefficients of this set of measures ranged from 0.71 to 0.88.

#### Phonological Awareness in English

We measured English PA using the *sound oddity task* (Bradley and Bryant, 1983; James, 1996; Li et al., 2012) and same/different judgment task (Treiman and Zukowski, 1991). Both tasks were designed to test all of the three phonological levels: syllable, onset-rime, and phoneme.

The *sound oddity task* was adapted from James (1996) and Li et al. (2012). On each trial, children heard three words from an audio CD; the trios were constructed so that exactly two of the three words shared an initial phoneme (e.g., *bus*, *bun*, *rug*), a medial phoneme (e.g., *bun*, *gun, pin*), or a final phoneme (e.g., *hop*, *top, doll*). Participants were asked to identify the word with the mismatching phoneme. Participants made their response by circling the word on a response sheet in which the corresponding grapheme of the tested phonemes was removed (e.g., _us, _un, _ug for *bus*, *bun*, *rug*). Practice trials were used to make sure the students understood the task. This task included 30 trios of words and took 1 min. The reliability was 0.90.

In the *same/different judgment task*, children were required to judge whether two words share a sound or not. The experimenter sounded out a pair of two spoken words that shared a sound at the beginning syllable (*hammer*, *hammock*), onset (*broom*, *brand*), or initial phonemes (*steak*, *sponge*), or at the shared final syllable (*compete*, *repeat*), shared rime (*spit*, *wit*), or shared final phonemes (*smoke*, *tack*). There were 10 word pairs for each of the six types mentioned above (60 total) and 80 word pairs that did not share a sound. It took students 3 min to complete this task. Reliability coefficients ranged from 0.86 to 0.89.

#### Orthographic Awareness in English

We used the Orthographic-Receptive Coding and Orthographic-Expressive Coding tasks (Berninger et al., 2010). For the receptive coding task, the children were exposed to either a real word (e.g., *word*) or a pseudoword (e.g., *wirf*) for 3 s, after which the word was removed from view. Children then had to judge whether the word (a) exactly matched a subsequently presented word (e.g., *werd* or *wirf*), (b) contained a given letter (e.g., *o* or *i*), or (c) contained a given letter group in exactly the same order (e.g., *ow* or *ir*). Stimulus items were designed so that correct answers could not be based solely on phonology but required attention to letters that had no phonological equivalent or that had alternative pronunciations. There were 30 sets of testing items in total. It took 3 min to complete this task. Reliability coefficients ranged from 0.70 to 0.78 for this measure.

For the Orthographic-Expressive Coding task, similar to a dictation task, the children were required to code the written words or pseudowords into temporary memory and reproduce all or parts of them in written format. There were 10 items of each of three types of reproductions: the whole word (e.g., *wirf)*, a single letter in a designated position (e.g., the third letter in the word *last*), or multiple letters in designated positions (e.g., second and third letters in the word *last*). It took 5 min to administer this task. Reliability coefficients ranged from 0.81 to 0.85.

#### Frequency in Chinese and English

Three measures of Chinese word frequency were obtained, all from Chen and Shu (2001). These frequency values were highly correlated (*r* = 0.84 to *r* = 0.95), so we aggregated them by first *z*-scoring each measure to put them on a common scale and then averaging them. Doing so reduces the measure-specific variance associated with any particular measure of word frequency (Bollen, 1989). Similarly, for English frequency, we averaged^1^ the Kuèera–Francis norms (Kucera and Francis, 1967) and the SUBTLEX_US_ corpus (Brysbaert and New, 2009), which were also highly correlated (*r* = 0.89).

#### Lexical Decision in Chinese

To select materials for the lexical decision task, we randomly sampled 240 characters (40 from each grade level) from the curriculum, ensuring that the items were representative of the compound regularities and configurations of Chinese characters. The basic configurations include left–right (e.g., 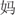), top–down (e.g., 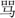), and outside–inside (e.g., 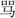). We defined characters as *high consistency* if the semantic radical appeared with the same pronunciation in more than 50% of characters (Shu and Anderson, 1999) and *low* if not, and we used the curricular grade level as a proxy for AoA. Another 240 pseudo-characters were created by adding, deleting, or shifting one stroke from the radicals within a legal character. The children received a practice trial to familiarize themselves with the task and then moved on the real testing session, in which they indicated whether each of the 480 characters was a real character or not, one a time; RT and accuracy were recorded by the computer.

#### Lexical Decision in English

To select materials for the lexical task in English, we randomly sampled 240 words (40 from each grade level) from the curriculum, ensuring that the testing items were representative of the letter–sound consistency, frequency of English words, and word reading level from each of six grades. Again, we took the curricular grade level as a proxy for AoA. Another 240 pseudo-characters were created by changing the onset, syllable, or rime of the real words; by swapping the letter orders within a word; or by changing a single letter or a cluster of letters within a word. The children received a practice trial to familiarize themselves with the task and then moved on to the real testing session, in which they judged whether each of the 480 words was real or not, one at a time; RT and accuracy were recorded by the computer.

Table 1 summarizes the descriptive statistics of all the variables.

**TABLE 1:** Means and (in parentheses) standard deviations of all Chinese and English measures among all readers in each of six grades.

**Measures**		**Grade 1**	**Grade 2**	**Grade 3**	**Grade 4**	**Grade 5**	**Grade 6**
**Chinese**							
*N*		137	111	121	115	123	114
PA	Total	14.1 (8.5)	14.7 (10.1)	16.0 (2.5)	16.8 (3.2)	17.1 (3.1)	19.2 (10.0)
OA	Total	18.8 (7.4)	21.0 (6.2)	24.0 (4.0)	25.1 (4.5)	27.0 (3.1)	27.9 (4.1)
Lexical decision	Accuracy	49%(20%)	50%(14%)	50%(9%)	55%(9%)	55%(8%)	56%(7%)
	RT (ms)	1,662(544)	1,588(360)	1,290(288)	1,371(211)	1,225(161)	1,081(228)
**English**							
*N*		137	111	121	115	123	114
PA	Total	14.0 (8.8)	14.5 (10.6)	15.7 (3.9)	16.8 (3.9)	17.4 (4.2)	19.1 (10.4)
OA	Total	18.6 (7.7)	20.8 (6.2)	23.9 (4.9)	25.3 (5.1)	26.7 (3.9)	28.1 (5.1)
Lexical decision	Accuracy	49%(20%)	50%(14%)	50%(9%)	55%(8%)	57%(7%)	58%(6%)
	RT (ms)	1,631(501)	1,409(338)	1,015(238)	1,021(75)	937 (161)	833 (197)

*PA, phonological awareness; OA, orthographic awareness; RT, response time.*

### Procedure

Participants completed all tasks in groups in their classrooms. The lexical decision tasks in both Chinese and English (20 min) were computerized, whereas all of the tasks assessing OA (20 min) and PA (15 min) were on paper. Across classrooms, we counterbalanced whether the computerized or paper tasks were presented first; the paper–pencil tasks were further counterbalanced in order. The tasks were later scored by two research assistants who had designed or familiarized with the tests; their inter-rater reliability was acceptable (all Pearson correlations above 0.85).

### Analytic Strategy

We analyzed our data using LME models (Baayen et al., 2008; Davies et al., 2017), which can simultaneously account for both participant- and item-level differences. In mixed-effects models, the unit of analysis is the outcome of an individual trial rather than the average across multiple trials. We examined two dependent measures: (a) the accuracy of lexical decision, using a generalized mixed-effects model as the log odds (*logit*) of correctly judging a word, and (b) the RT (in ms) for correct lexical decisions, log-transformed to reduce positive skew.

Our fixed effects of interest included, at the item level, frequency, radical consistency (for Chinese only), and curricular grade level, and at the subject level, PA and OA. A further goal was to examine the interactions of pupil and character properties across age from grades 1 to 6. Thus, we allowed each of the effects named above to vary both linearly (i.e., a steady increase or decreases from grades 1 to 6) and quadratically (i.e., an effect strongest or weakest in the middle grades). Finally, because there is some evidence that, at least in English, frequency effects vary with reading skill (e.g., Perfetti and Hogaboam, 1975), we allowed the frequency effect to interact with our two measures of reading skill: PA and OA. We included only these interactions, for which we had *a priori* hypotheses; to avoid a combinatorial explosion of interaction terms given our large number of predictors, we did not include any higher-order interactions or other two-way interactions. Because all of our predictors except grade level were on arbitrary scales, we centered and *z*-scored them to facilitate comparison across variables. All variables (including grade level) were mean-centered to produce estimates of main effects averaging across the other variables, analogous to those from an ANOVA.

In all models, we included both participant, classroom, and item (word) random intercepts^2^ to account for both participant differences and, critical to the motivation of the analysis, item differences. We adopted a model-based approach to outlier detection by fitting an initial model, eliminating observations with residuals more than three standard deviations from the mean, and then refitting each model (Baayen, 2008). This procedure identifies observations that are outliers after considering all fixed and random effects of interest.

All models were fit in R using package *lme4* (Bates et al., 2015). Fixed effects were tested using the Wald *z* test for logit models and the Sattherthwaite approximation to the *t* distribution for Gaussian models (package *lmerTest*; Kuznetsova et al., 2017), all with an α = 0.05 criterion for significance.

## Results

### Overall Grade-Level Differences

**FIGURE 1:**
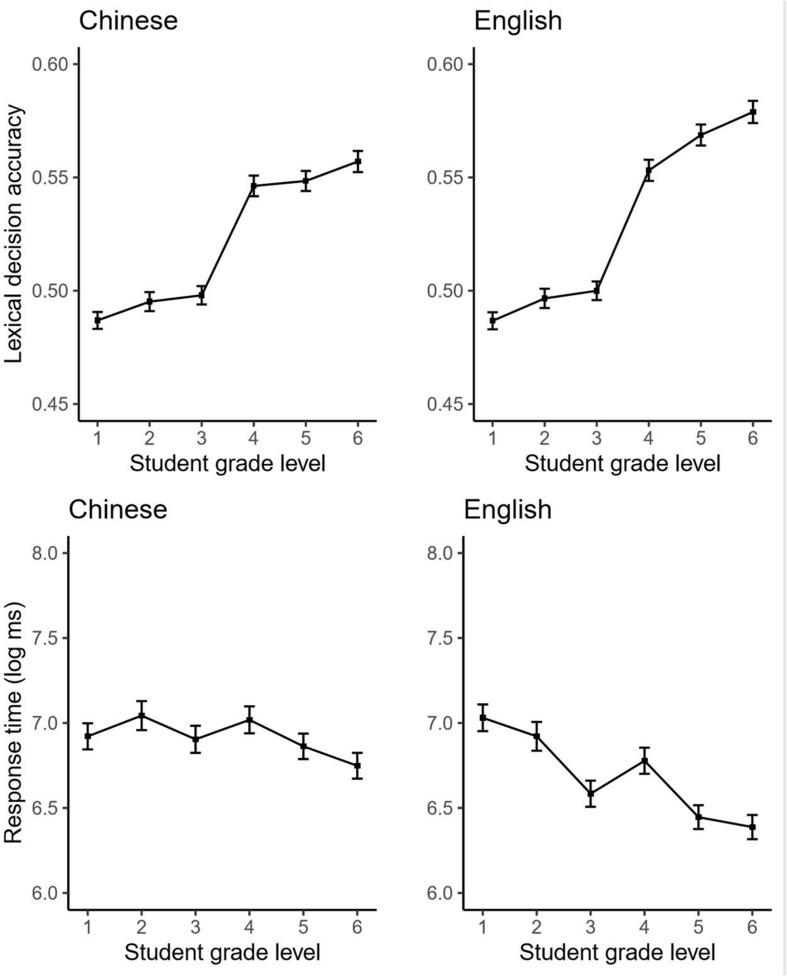
Proportion accuracy for lexical decisions (top panels) and response time for correct lexical decisions (bottom panels) as a function of student grade level in both Chinese (left panels) and English (right panels). Error bars indicate 95% confidence intervals computed across subjects.

We first examine average performance from grade 1 to grade 6 in reduced models that included only student grade level. These models allow us to describe the overall pattern of grade-level differences, setting aside any individual differences (e.g., Peng et al., 2019), and to compare Chinese and English directly by including all observations with language as an additional predictor variable. Table 2 and the top panel of Figure 1 display these overall developmental differences with fewer than 0.1% of outlying observations removed. Overall performance did not significantly differ across languages, *p* = 0.50, and was close to 50%; because this was neither at floor nor ceiling, it allowed us ample room to detect effects of our variables of interest.

**TABLE 2:** Fixed-effect estimates from mixed-effects logit model of lexical decision accuracy.

	**Estimate**	**SE**	**Wald *z***	***p***
Intercept (baseline log odds of accuracy)	0.156	0.061	2.54	0.01
Language (English vs. Chinese)	0.022	0.086	0.26	0.80
Student grade level—linear effect	0.103	0.016	6.33	<0.001
Student grade level—quadratic effect	0.004	0.011	0.39	0.69
Language × student grade level—linear effect	0.021	0.003	6.13	<0.001
Language × student grade level—quadratic effect	0.005	0.002	2.27	0.02

Nevertheless, lexical decision accuracy increased from grade 1 to grade 6, as reflected by the significant linear effect of grade level. Further, a positive language × linear grade interaction indicated that this increase was especially steep for English. Lastly, a language × quadratic grade interaction indicates some departure from a linear growth rate for English.

Indeed, inspection of the means suggests an especially sharp, non-linear increase between grades 3 and 4. *Post hoc* tests using the Tukey correction for multiple comparisons (R package *emmeans*; Lenth, 2019) confirmed that this growth from grade 3 to grade 4 was the only significant year-to-year difference, in both Chinese (*p* < 0.05, all other *p*s ≥ 0.95) and English (*p* < 0.05, all other *p*s ≥ 0.94).

The bottom panel of Figure 1 displays the grade-level differences for RTs to correct lexical decisions (180,231 trials for Chinese and 179,370 for English), and Table 3 the results from the mixed-effects model with 0.8% of outlying RTs removed. Overall, RTs declined (i.e., became faster) from grade 1 to grade 6. Unlike for accuracy, there was also a main effect of language, with English words being responded to more quickly than Chinese. Further, interactions with grade level indicated that this difference increased over time; RTs declined more quickly for English than for Chinese (linear term), although this change eventually leveled off (quadratic term).

**TABLE 3:** Fixed-effect estimates from mixed-effects logit model of response time for accurate lexical decisions.

	**Estimate**	**SE**	***t***	***df***	***p***
Intercept (baseline log RT)	6.846	0.048	142	16.58	<0.001
Language (English vs. Chinese)	−0.263	0.017	−15.19	979	<0.001
Student grade level—linear effect	−0.085	0.017	−4.92	15.78	<0.001
Student grade level—quadratic effect	−0.007	0.012	−0.63	15.71	0.54
Language × student grade level—linear effect	−0.086	0.001	−77.6	>355,000	<0.001
Language × student grade level—quadratic effect	0.026	0.001	34.8	>355,000	<0.001

### Effects of Item-Level Variables

#### Accuracy

**FIGURE 2:**
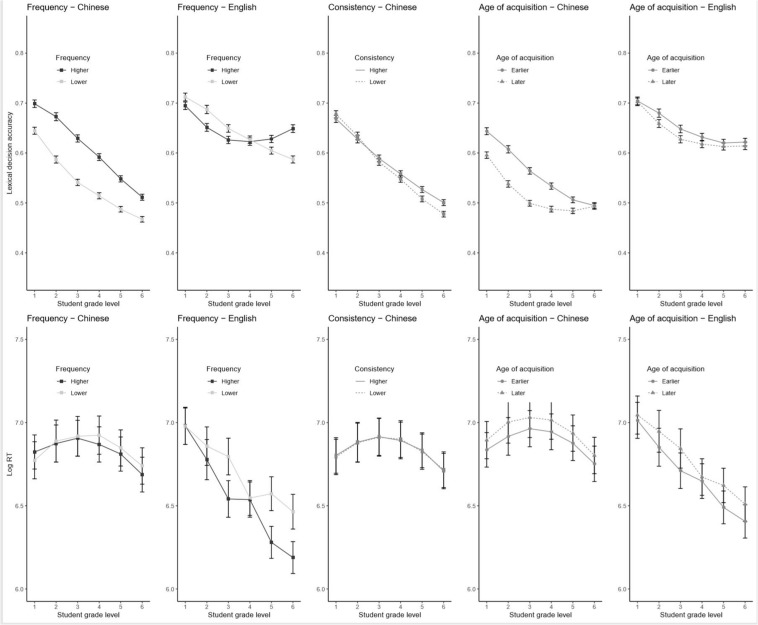
Model-predicted proportion accuracy for lexical decisions (top panels) and response time for correct lexical decisions (bottom panels) as a function of the partial effects of student grade level and item-level properties. Frequency, consistency, and age of acquisition are depicted as median splits for purposes of visualization but were entered as continuous variables into the mixed-effects models. Error bars depict 95% confidence intervals across subjects.

Next, we fit our main models including all of the item-level and subject-level variables of interest. Here, we fit models for Chinese and English separately because we had slightly different sets of predictors for the two languages (i.e., our measure of consistency was not generalizable to English). Table 4 displays the results from the models of accuracy in Chinese and English with fewer than 0.01% of outlying observations removed from each model, and Figure 2 plots model-predicted partial effects (via R package *remef*; Hohenstein and Kliegl, 2020) for each variable of interest.

**TABLE 4:** Fixed-effect estimates from mixed-effects logit model of lexical decision accuracy for Chinese (top panel) and English (bottom panel) as a function of item- and student-level variables.

	**Estimate**	**SE**	**Wald *z***	***p***
**Chinese**				
Intercept (baseline log odds of accuracy)	0.338	0.180	1.88	0.06
Student grade level—linear effect	−0.205	0.018	−11.52	<0.001
Student grade level—quadratic effect	0.015	0.012	1.28	0.20
**Item-level variables**				
Frequency	0.222	0.085	2.62	0.01
Frequency × linear student grade level	−0.010	0.004	−2.72	0.01
Frequency × quadratic student grade level	−0.012	0.002	−5.32	<0.001
Consistency	0.017	0.064	0.27	0.79
Consistency × linear student grade level	0.021	0.002	8.47	<0.001
Consistency × quadratic student grade level	<0.001	0.002	−0.06	0.95
Curricular grade level	−0.070	0.047	−1.50	0.13
Curricular grade level × linear student grade level	0.017	0.002	9.35	<0.001
Curricular grade level × quadratic student grade	0.005	0.001	3.99	<0.001
**Student-level variables**				
Phonological awareness	0.165	0.043	3.81	<0.001
Phonological awareness × linear student grade level	−0.014	0.010	−1.41	0.16
Phonological awareness × quadratic grade level	−0.016	0.009	−1.85	0.06
Orthographic awareness	0.599	0.036	16.63	<0.001
Orthographic awareness × linear student grade level	−0.034	0.013	−2.58	0.01
Orthographic awareness × quadratic grade level	0.022	0.009	2.61	0.01
Frequency × phonological awareness	−0.052	0.006	−8.19	<0.001
Frequency × orthographic awareness	−0.020	0.005	−3.77	<0.001
English				
Intercept (baseline log odds of accuracy)	0.645	0.137	4.71	<0.001
Student grade level—linear effect	−0.116	0.017	−6.66	<0.001
Student grade level—quadratic effect	0.030	0.011	2.59	0.01
**Item-level variables**				
Frequency	−0.035	0.060	−0.58	0.56
Frequency × linear student grade level	0.050	0.003	16.79	<0.001
Frequency × quadratic student grade level	0.014	0.002	8.13	<0.001
Curricular grade level	−0.144	0.034	−4.30	<0.001
Curricular grade level × linear student grade level	0.015	0.001	10.44	<0.001
Curricular grade level × quadratic student grade	0.005	0.001	4.84	<0.001
**Student-level variables**				
Phonological awareness	0.230	0.040	5.77	<0.001
Phonological awareness × linear student grade level	−0.012	0.010	−1.20	0.24
Phonological awareness × quadratic grade level	−0.027	0.009	−3.14	<0.01
Orthographic awareness	0.396	0.034	11.53	<0.001
Orthographic awareness × linear student grade level	−0.089	0.012	−7.27	<0.001
Orthographic awareness × quadratic grade level	0.026	0.008	3.13	<0.01
Frequency × phonological awareness	−0.005	0.005	−1.01	0.31
Frequency × orthographic awareness	> −0.001	0.006	−0.07	0.95

We first turn our attention to the effect of item-level variables on lexical decision accuracy. The effect of word frequency (upper-left panels of Figure 2) showed different patterns of grade-level differences across languages: In Chinese, more frequent words were responded to more accurately across grade levels, but this effect diminished somewhat in higher grades as the less frequent words “caught up” in accuracy to the higher-frequency words. By contrast, in English, the overall main effect of word frequency was not significant; in early grades, lower-frequency words were actually recognized *better*, and a beneficial effect of word frequency emerged only in grade 5 and above.

Further, in Chinese, the word frequency effect in accuracy was qualified by interactions with both orthographic and PA such that word frequency was less important for higher-skilled readers; there were no such interactions in English. Note, however, that the standardized parameter estimates for the interactions were of substantially smaller magnitude than the main effect of frequency; that is, the frequency effect was reduced for readers of higher skill but not eliminated.

The effect of consistency in Chinese words (upper-middle panel of Figure 2) varied linearly across grade levels. At lower grades, low-consistency words were responded to slightly more accurately than high-consistency words, but this reversed over time such that high-consistency words were eventually judged more accurately.

Lastly, words with earlier AoA were generally responded to more accurately (upper-right panels of Figure 2). AoA did not have a significant main effect on accuracy in Chinese but interacted with student grade level such that the benefit of AoA was evident most strongly in earlier grades. By comparison, in English, the benefit of word AoA was strongest in middle grades, and the main effect of AoA was also significant across grades.

#### Response Time

Next, we turn to how these same variables affected RTs in correct lexical decision trials. Table 5 displays the results of these models, with 0.8% and 1.1% of outlying RTs removed in Chinese and English, respectively.

**TABLE 5:** Fixed-effect estimates from mixed-effects logit model of response time for accurate lexical decisions for Chinese (top panel) and English (bottom panel) as a function of item- and student-level variables.

	**Estimate**	**SE**	***t***	***df***	***p***
**Chinese**					
Intercept (baseline log RT)	6.912	0.064	108	28.44	<0.001
Student grade level—linear effect	−0.014	0.021	−0.69	17.79	0.50
Student grade level—quadratic effect	−0.025	0.014	−1.78	16.39	0.09
**Item-level variables**					
Frequency	−0.024	0.016	−1.52	493	0.13
Frequency × linear student grade level	−0.011	0.001	−9.62	>175,000	<0.001
Frequency × quadratic student grade level	0.004	0.001	5.75	>175,000	<0.001
Consistency	<0.001	0.012	0.01	490	0.99
Consistency × linear student grade level	−0.001	0.001	−0.96	>175,000	0.34
Consistency × quadratic student grade level	0.001	0.001	0.50	>175,000	0.62
Curricular grade level	0.024	0.009	2.70	491	0.01
Curricular grade level × linear student grade level	> −0.001	0.001	−0.20	>175,000	0.84
Curricular grade level × quadratic student grade	−0.001	0.001	−2.15	>175,000	0.03
**Student-level variables**					
Phonological awareness	−0.023	0.030	−0.76	703	0.45
Phonological awareness × linear student grade level	0.023	0.007	3.48	708	0.001
Phonological awareness × quadratic grade level	−0.002	0.006	−0.27	705	0.79
Orthographic awareness	−0.058	0.025	−2.33	709	0.02
Orthographic awareness × linear student grade level	> −0.001	0.009	−0.05	703	0.96
Orthographic awareness × quadratic grade level	−0.001	0.006	−0.21	712	0.84
Frequency × phonological awareness	<0.001	0.002	−0.27	>175,000	0.79
Frequency × orthographic awareness	0.004	0.002	2.26	>175,000	0.02
**English**					
Intercept (baseline log RT)	6.614	0.053	124	26.91	<0.001
Student grade level—linear effect	−0.130	0.017	−7.55	17.10	<0.001
Student grade level—quadratic effect	0.008	0.012	0.66	16.56	0.52
**Item-level variables**					
Frequency	−0.102	0.012	−8.61	488	<0.001
Frequency × linear student grade level	−0.032	0.001	−34.19	>175,000	<0.001
Frequency × quadratic student grade level	<0.001	0.001	0.81	>175,000	0.42
Curricular grade level	0.032	0.007	4.76	488	<0.001
Curricular grade level × linear student grade level	0.004	<0.001	9.32	>175,000	<0.001
Curricular grade level × quadratic student grade	−0.001	<0.001	−3.31	>175,000	<0.01
**Student-level variables**					
Phonological awareness	−0.048	0.016	−3.02	695	<0.01
Phonological awareness × linear student grade level	0.014	0.004	3.53	699	<0.001
Phonological awareness × quadratic grade level	0.005	0.003	1.42	698	0.16
Orthographic awareness	−0.021	0.014	−1.50	700	0.14
Orthographic awareness × linear student grade level	0.006	0.005	1.16	695	0.25
Orthographic awareness × quadratic grade level	−0.045	0.003	−1.29	703	0.20
Frequency × phonological awareness	<0.001	0.002	0.09	>175,000	0.93
Frequency × orthographic awareness	−0.007	0.002	−3.56	>175,000	<0.001

Word frequency (lower-left panels of Figure 2) did not have a significant main effect on RTs in Chinese; there was, however, a significant developmental trend such that a frequency effect began to emerge in higher grades. By contrast, frequency had a facilitatory effect on RTs across grade levels in English, and this frequency difference increased with grade level as recognition of high-frequency words especially accelerated.

The frequency effect in Chinese was qualified by an interaction with OA such that frequency speeded responding more for students with poor OA; again, however, this interaction was of relatively small magnitude such that OA modulated but did not eliminate the frequency effect. The English frequency was also qualified by an interaction but in the opposite direction: Students with higher OA in English showed a *larger* frequency effect.

Radical consistency (lower-middle panel of Figure 2) had no effects on RTs. Curricular grade level (lower-right panels of Figure 2) had significant main effects in both Chinese and English such that words with earlier AoA were responded to more quickly across grade levels. For Chinese, a significant quadratic trend indicated that this effect was largest in the middle grades, whereas for English, the effect became larger beyond the first grade.

#### Summary

Word frequency facilitated both the accuracy and speed of lexical decision but showed different patterns of grade-level differences across languages. The benefit of frequency on accuracy diminished with grade level in Chinese but *increased* over time in English. Nevertheless, in both languages, the benefit on RTs was largest in later grades.

The benefit of frequency was especially large for students with poor PA or OA in Chinese, whereas in English, frequency was more beneficial for students with *higher* OA.

Even when controlling for word frequency, words learned earlier in the curriculum (i.e., earlier AoA) were generally responded to more quickly and accurately. Similar to frequency, this effect was stronger in earlier grades in Chinese but stronger in later grades for English. Lastly, the consistency of Chinese radicals did not affect RT, but it did have varying effects on response accuracy, such that high-consistency words were initially responded to less accurately but, in later grades, more accurately.

### Effects of Student-Level Variables

#### Accuracy

**FIGURE 3:**
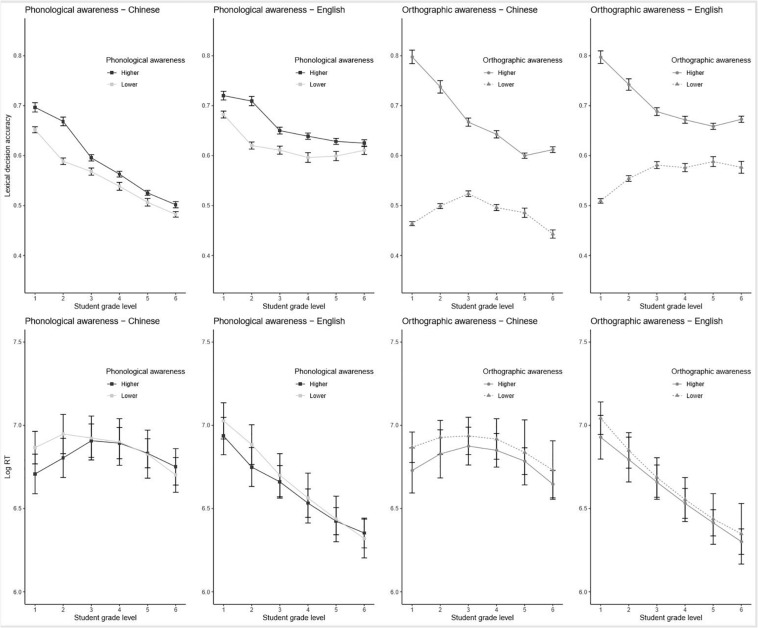
Model-predicted proportion accuracy for lexical decisions (top panels) and response time for correct lexical decisions (bottom panels) as a function of the partial effects of student grade level and subject-level properties. Phonological and orthographic awareness are depicted as median splits within each grade level for purposes of visualization but were entered as continuous variables into the mixed-effects models. Error bars depict 95% confidence intervals across subjects.

To analyze the student-level variables, we first return to Table 4 to consider their effect on accuracy. PA (upper-left panels of Figure 3) had a main effect on accuracy in both languages such that students with greater PA responded substantially more accurately; in both languages, this effect was largest in the early grades.

The effect of OA on accuracy (upper-right panels of Figure 3) was even more similar across languages. Students with greater OA responded more accurately, but there were significant linear and quadratic developmental trends in both languages, such that the effect of OA was largest in the earlier grades, smallest in the middle grades, and moderately sized in the upper grades.

Recall, further, that the benefits of OA and PA in Chinese were qualified by an interaction with word frequency such that OA and PA were most beneficial for lower-frequency words. Nevertheless, the standardized estimate for this interaction was small relative to the main effects of PA and OA; thus, PA and OA were helpful even for judging high-frequency words.

#### Response Time

In contrast to accuracy, PA did not have a reliable main effect on RT in Chinese (lower-left panels of Figure 3). However, there was a significant linear trend; PA benefited RT in earlier grades, but this effect disappeared over time. In L2 English, there was a significant main effect, but this effect nevertheless declined over time as well.

For OA (lower-right panels of Figure 3), there was a significant facilitatory main effect across grade levels in Chinese but no significant effects on RT in L2 English.

#### Summary

PA and OA had more robust effects on accuracy than RT. The developmental trend of these effects was similar across languages such that these abilities most benefited performance in the earlier grades and showed diminished effects in the higher grades. OA benefited both accuracy and RT in Chinese (with the benefit to accuracy again being largest in the earliest grades) but benefited only accuracy in L2 English.

The benefits of orthographic and PA in Chinese were stronger for lower-frequency words; that is, good PA and good OA could help compensate for the difficulty associated with reading low-frequency words.

## Discussion

In this current study, we explored the general development of word recognition development across grades in L1 Chinese and L2 English, as well as how these grade-level differences are influenced by both item- and subject-level characteristics. Using the lexical decision task, we assessed word recognition of 240 Chinese characters and 240 English words cross-sectionally from grade 1 to grade 6. We used LME modeling to simultaneously consider item-level (frequency, consistency, and curricular grade level) and subject-level (OA and PA) variables.

Three major findings were obtained. First, as grade level increases, accuracy increases and RT speeds up for both English and Chinese. In particular, it seems that the transition from grade 3 to grade 4 (with students’ age between 10 and 11 years old) is a period when accuracy in word recognition sharply increases Second, word frequency and curricular grade level each predict word recognition in both languages but develop differently across grades, with the benefits of word frequency stronger in early grades in L1 Chinese but in later grades (i.e., grade 4 and above) in L2 English. The benefit of consistency of Chinese characters also increased with students’ age from grade 1 to grade 6. Third, we observed item-by-subject interactions in Chinese such that both PA and OA were more beneficial to low-frequency words in accuracy; OA was also more beneficial to low-frequency words in RT. We did not observe this interaction in L2 English; if anything, OA was more beneficial for high-frequency words in L2 English.

We discuss these major results first in terms of our statistical approach. We then turn to the item-level and subject-level effects and their interaction effects and what these effects indicate about the development of word recognition. Finally, we provide some consideration of how theoretical models of reading development generalize to a cross-linguistic perspective on word recognition.

### Mixed Linear Modeling of Cross-Linguistic Developmental Data

The development of multilevel LME models permits a closer look at word recognition development through item-level analysis of word reading (e.g., Gilbert et al., 2011; Steacy et al., 2016; Guan et al., 2020). Here, we applied such models to understanding the development of word recognition from a cross-linguistic perspective. Similar to the growth curve analyses conducted in previous research (Berninger et al., 2010, 2013; Goswami, 2010), we examined how word recognition changed between grades 1 and 6—were they steady linear changes, or did they show asymptotic or other non-linear changes?

At the broadest level, the models showed similar and generalizable patterns of word learning development across languages, i.e., as grade level increases, the recognition accuracy increases and RT speeds up for both English and Chinese. In particular, for both L1 Chinese and L2 English, the recognition accuracy increased sharply from grade 3 to grade 4 but plateaued afterward.

A particular contribution of this current study is the use of mixed effects to simultaneously examine not only item- and subject-level effects but also their interactions (and for both L1 Chinese and L2 English). We discuss those effects more in detail below.

### Item-Level Effects

We found that two item-level variables—word frequency and AoA (operationalized here as curricular grade level)—were beneficial in both languages. Further, AoA showed similar grade-level differences across languages such that it diminished with advancing grade levels. Nevertheless, frequency showed somewhat different patterns across languages: In L1 Chinese, the benefit of high frequency diminished with grade level, but in L2 English, high-frequency words were initially judged less accurately, and frequency only became beneficial later.

It is noteworthy that, in general, these item-level effects decreased with age. Murray and Forster (2004) had argued that the frequency effect in lexical access or word recognition should not change along with growing overall experience. However, later, based on findings from a range of methods, Davies et al. (2017) suggested that word frequency and AoA effects decline with increasing age. That is, as readers grow older and gain more experience, their performance is less affected by how common the words are in the language or by the time point at which they learnt the words. This is likely because readers in more advanced grades have encountered more of these words and thus can handle them all more accurately. Our results support this latter claim.

Within L1 Chinese, we also examined a third item-level variable: radical consistency. For this variable, we found that high consistency was associated with superior recognition in later grades but poorer performance in earlier grades. Previous literature has not provided a clear picture on the development of this consistency effect, because grade levels have been sampled somewhat sporadically. For example, Yang and Peng (1997) tested third- and sixth-grade school children in a naming task and found that both showed a consistency effect (as defined in Fang et al., 1986). Shu and Wu (2006) replicated the experiment of Yang and Peng (1997) with fourth- and sixth-grade children and found that both showed consistency effects. Shu et al. (2000) found that this effect grew stronger as children got older. Shu et al. (2003) have also found that children need a long time to develop phonetic consistency awareness. Our results are also consistent with this claim in that we found that consistency was only beneficial in later grades.

Taken together, our results suggest continuous development of word learning in both Chinese and English. The developmental patterns begin at an earlier age in L1 Chinese and at a later age in L2 English. A plausible interpretation is that the effects of word features like frequency and consistency begin to manifest after the learners have grasped some basic awareness and knowledge of word-level skills—at middle grades (e.g., grade 3) in L1 Chinese and advanced grades in L2 English (e.g., grades 5 and 6), since English is introduced in formal classroom instruction after grade 3 (NIES, 2012). Interestingly, these item-level effects may interact with subject-level effects, which we discuss below.

### Subject-Level Effects

The subject-level effects suggest a general benefit of PA and OA in word recognition, though mainly in response accuracy rather than RT. The benefits of these skills were largest in earlier grades, when beginning readers may not yet have other applicable skills or knowledge. These findings are consistent with prior work, so the subject-level effects alone are not a major focus in the current study.

### Interaction Effects

Of greater interest was how the subject-level factors moderated the strength of item-level effects. PA and OA interacted with character frequency in L1 Chinese to affect response accuracy, and in the case of OA, it interacted with character frequency to affect RT. Specifically, readers with lower PA and OA benefited more from character frequency, whereas readers with high skill could handle even low-frequency characters in Chinese. To put it another way, reading skill mattered more when reading low-frequency characters than high-frequency ones. This is consistent with past evidence that frequency effects are generally larger for less-skilled readers (e.g., Perfetti and Hogaboam, 1975; Davies et al., 2017); here, we show that these effects extend to developing L1 Chinese readers.

In contrast, there were no frequency × PA interactions in L2 English, and the frequency × OA interaction was reversed such that students with higher OA in English showed a larger frequency effect. We suspect that this might be due to the fact that language experience differs between Chinese and English in our sample. In this study, we recruited students who were beginning learners of English as an L2, i.e., they were not balanced Chinese–English bilinguals. These students were just beginning to accumulate their language experience, such that only those students with higher OA may have been able to capitalize on word frequency. That is, even those students relatively high in L2 English OA may have only had a level of reading ability equal to what constituted “poor” OA in L1 Chinese.

### A Theoretical Model of Reading Development Generalizable Across Languages

The theoretical model of Zevin and Seidenberg (2002) predicts that effects of consistency, frequency, and word AoA vary over time. As readers accumulate experience, their initial experiences (i.e., AoA) matter less, and their performance becomes instead dominated by more general regularities of the orthography-to-phonology mapping. Although our goal was not to conduct a global and complete test of this model, we at least provide supportive evidence by showing that (a) AoA effects diminish across grades, whereas (b) effects of a radical’s phonetic consistency become larger.

The interactions of age with frequency or AoA are consistent with a gradual ceiling effect predicted to result from the assumption—inherent in connectionist network systems—of asymptotic learning based on distributed representations and a non-linear input–output function (Van Orden et al., 1990; Plaut et al., 1996). That is to say, the effects of psycholinguistic properties change as a function of the oral reading system, approaching maximal efficiency as experience accumulates and skill develops. Another example of this principle is that, while the consistency effect in English influences children’s reading (Laxon et al., 1988, 2002), it is smaller for more skilled readers (Laxon et al., 1988). This is because the other reading component skills, such as PA or OA, develop and compensate for difficult words. We observed similar effects in our study insofar as frequency effects were weaker in L1 Chinese for readers high in PA and/or OA.

This principle of asymptotic word learning applies cross-linguistically in both L1 Chinese and L2 English. For instance, in the present study, we found that AoA effects diminished with grade level increases in both L1 Chinese and L2 English. Indeed, these features of connectionist reading models can apply to all languages and any type of script provided that the statistical constraints of a specific language are known beforehand.

### Future Directions

In this study, we conducted a cross-sectional comparison of grades 1 and 6. At an empirical level, future studies could examine the developmental patterns of cross-linguistic word learning across even broader sections of the life span and could collect longitudinal, rather than cross-sectional, data. Davies et al. (2017) argue that frequency effects change with age, most principally in the transition from childhood into adulthood. In their item-level analysis, the frequency effect was larger in children’s RTs than in young adults.’ In their subject-level analyses, the per-subject estimates of the frequency effect coefficient varied in relation to age, but the age effect on frequency coefficients was curvilinear; it appeared to be stronger for younger children.

At a technical level, we encourage future researchers to consider the use of an LME model to assess word learning and reading development across the life span. Researchers have typically focused either on the effects of word properties in item-level analyses or on the effects of individual differences in subject-level analyses. The benefit of a multilevel analysis of reading, such as ours, is that it allowed for the examination of item-by-subject interactions. One insight from this approach is that the psycholinguistic effects of Chinese characters on the development of literacy systematically vary in relation to individual differences in age and reading ability of a pupil. Second, variation in stimulus properties emerges against a backdrop of large, overarching, effects on performance due to individual differences. Mixed-effects models show that the effects of word properties, and their modulation by individual differences, are significant, but that the dominant source of variance in reading performance is those individual differences (see Davies et al., 2017).

Lastly, more comparable language-specific measures for both Chinese and English should be designed and validated. We analyzed Chinese and English in separate models because we did not have a comparable measure of one item-level variable, consistency, for English, which would have allowed us to directly compare languages within a single model. Determining English consistency would require hand calculation (e.g., Weekes et al., 2006); this was outside the scope of the current study but could be conducted in the future for more comparable models. There were also some limitations in the measures we did obtain. For instance, our expressive coding task in English also required children to hold material in working memory, so variation in these scores might reflect memory skills as well as orthographic skills. Similarly, one of our Chinese OA tasks, radical knowledge, could potentially be solved on the basis of visual analysis alone—but note that this was not true of the other task measuring OA in Chinese, stroke awareness.

## Conclusion

Our study shows the importance of both stimulus-related, item-level (exogenous), and individual-related, child-level (endogenous), psycholinguistic factors in learning to recognize words. First, we found similar trends for word reading development in both L1 Chinese and L2 English in a cross-sectional comparison of Chinese elementary students from grades 1 to 6, and we assume that this serves as a proxy for age-related effects. Second, and most importantly, we contribute evidence that the constraints on acquisition of literacy in Chinese as an L1 and English as an L2 are multifaceted and include exogenous (stimulus-related) properties as well as endogenous (subject-related) properties. We conclude that these properties interact to produce literacy in Chinese and English and form the generalizable basis of a theoretical view of early-years reading from the cross-linguistic perspective.

## Data Availability Statement

The datasets generated for this study are available on request to the corresponding author.

## Ethics Statement

The studies involving human participants were reviewed and approved by the University of Science and Technology Beijing. Written informed consent to participate in this study was provided by the participants’ legal guardian/next of kin.

## Author Contributions

CG conceived, designed, and performed the experiments. CG and SF analyzed the data and wrote the manuscript.

## Conflict of Interest

The authors declare that the research was conducted in the absence of any commercial or financial relationships that could be construed as a potential conflict of interest.

## Footnotes

1 We discovered after data collection that 10 of our 480 English words (2%) did not have word frequency information available in the SUBTLEX_US_ corpus; these items were eliminated from analysis.

2 We did also consider models with random slopes, but the model estimation process failed to converge. However, qualitative inspection of the parameter estimates from these models suggests that, had the random slopes been included, the principal conclusions would be unchanged.
